# The value of non-contrast and contrast-enhanced time-of-flight magnetic resonance angiography in the follow-up assessment of cerebral aneurysms treated with flow diverter stents

**DOI:** 10.1007/s00234-026-03996-9

**Published:** 2026-04-14

**Authors:** Amir Reza Rezaeiserej, Patrick Thurner, Gergely Bertalan, Maté Czencz, Miklós Krepuska, Anna A. Kyselyova, Ramona Alexandra Todea, Jawid Madjidyar, Tilman Schubert, Nicolin Hainc, Zsolt Kulcsar

**Affiliations:** University Hospital of Zurich, Zurich, Switzerland

**Keywords:** Cerebral aneurysm, Flow diverter, TOF MRA, TOF-CE MRA, DSA

## Abstract

**Purpose:**

Magnetic resonance imaging follow-up of cerebral aneurysms treated with flow diverters (FD) has a crucial role in assessing changes of the aneurysm size, intra-aneurysmal thrombosis and of the peri-aneurysmal environment. The value of Time-of-Flight (TOF) Magnetic Resonance Angiography (MRA) and TOF-Contrast-Enhanced (TOF-CE) MRA in assessing residual aneurysms and in-stent stenosis remains debated. This study evaluates the diagnostic performance of these modalities compared with Digital Subtraction Angiography (DSA).

**Methods:**

A retrospective analysis of 73 patients treated with FD between 2018 and 2022 was conducted. Patients underwent TOF MRA, TOF-CE MRA and DSA imaging post-treatment and at one-year follow-up. Diagnostic accuracy, sensitivity and specificity of the two MRA modalities were calculated relative to DSA.

**Results:**

Compared to DSA, TOF MRA demonstrated an accuracy of 90% in detecting residual aneurysm filling, with a sensitivity of 96% and specificity of 66%, while TOF-CE MRA showed an accuracy of 88% in detecting residual aneurysms, with a sensitivity of 91% and specificity of 73%. Misclassification rates increased for larger aneurysms. Both modalities were limited in detecting in-stentstenosis and aneurysm occlusion.

**Conclusions:**

TOF MRA and TOF-CE MRA showed high sensitivity and moderate specificity in detecting residual aneurysm filling, but contrast material provided no significant added benefit. MRI cannot fully replace DSA for evaluating residual aneurysms and in-stent stenosis. The combination of DSA and MRA remains the most effective follow-up strategy at 12 months, with further MR-only follow-up feasible in most patients.

## Introduction

 The treatment of intracranial aneurysms has evolved significantly with the introduction of flow diverter stents (FD), promoting intrasaccular thrombosis, inducing aneurysm remodeling and healing [1, 2]. This approach has shown a high efficacy in aneurysm cure rates, with low morbidity and mortality [3, 4].

Post-treatment follow-up is critical to identify residual aneurysm filling or in-stent stenosis, which can impact patient outcomes [5, 6]. Digital Subtraction Angiography (DSA) remains the gold standard for imaging of aneurysm occlusion due to its high spatial resolution and capacity for detailed vascular visualization [7]. It is well known however, that partially or completely thrombosed aneurysms treated with FD, showing good angiographic results, may become unstable and increase in size, eventually leading to delayed ruptures [8]. Further, DSA is invasive, carries certain procedural risks, and involves radiation exposure, making it less desirable for routine follow-up [9].

For aneurysms treated with coil embolization, non-invasive alternatives such as Magnetic Resonance Angiography (MRA) are routinely used, particularly Time-of-Flight (TOF) MRA, which visualizes blood vessels based on flow dynamics without contrast agent [10]. Evidence from follow-up studies of coiled aneurysms supports its use [11], with high sensitivity and specificity for aneurysm occlusion or recurrence estimation [12].

Magnetic resonance imaging (MRI) has become an increasingly important tool in the follow-up of aneurysms treated with stent-assisted techniques and after flow diverter placement. The ability of MRI to visualize aneurysms without ionizing radiation makes it a favorable option for regular monitoring. Specifically, Time-of-Flight (TOF) MRA and TOF-CE MRA have been suggested as non-invasive alternatives to DSA for detecting residual aneurysm filling and in-stent stenosis. Recent studies have shown that TOF-MRA is effective in detecting aneurysm occlusion in the majority of cases, particularly in small aneurysms [13–15]. However, their efficacy in detecting residual aneurysms and in-stent stenosis in larger aneurysms remains uncertain, with some evidence indicating increased misclassification rates [15]. The artifacts arising from the implanted stents and FDs can severely disturb diagnostic imaging. Signal intensity is influenced not only by the orientation of the flow diverter within the static magnetic field and imaging plane, but also by device-related effects. The implant reduces intraluminal signal in the parent vessel and in the surrounding perivascular space as a result of radiofrequency shielding and magnetic susceptibility [16].

To increase signal and detect slow filling aneurysm residues, the administration of contrast agent might provide a better signal for assessing in-stent stenosis and residual aneurysms. Recent advancements in TOF-CE have further improved vessel contrast and visibility, making it a viable option for monitoring treated aneurysms [17], however data on its use in follow-up of FD treated lesions remains scarce.

This study aims to compare the diagnostic accuracy of TOF MRA and TOF-CE MRA with DSA in a cohort of patients who underwent flow-diverter treatment for aneurysms, assessed both post-treatment and at one-year follow-up. We hypothesize that contrast enhancement in TOF-CE MRA could provide better visualization of small residual aneurysms, which may not generate sufficient signal in standard TOF MRA. By examining these imaging modalities side-by-side and compared to DSA, we aim to clarify their roles in clinical practice, particularly focusing on the potential advantage of TOF-CE MRA in detecting very small residual aneurysm fillings that may not be visible in TOF MRA.

## Materials and methods

### Patients

A retrospective analysis was conducted on 143 patients with intracranial aneurysms who underwent endovascular treatment with flow diverter devices at our institution between 2018 and 2022. All patients were examined using a standardized imaging protocol that included both MRI and DSA at defined post-treatment intervals. The imaging protocol remained consistent across all cases and comprised non-contrast TOF MRA, TOF-CE MRA, and conventional DSA, allowing for a uniform comparison of diagnostic performance across modalities.

All patients were treated and followed up at a single academic neuroradiology center. As part of routine clinical care, a follow-up assessment was performed at one year after flow diverter implantation, including both MRI and DSA, to evaluate aneurysm occlusion status, in-stent stenosis, and treatment response.

**Inclusion criteria**

1. patient older than 18;
2. availability of TOF MRA and TOF-CE MRA ;
3. availability of DSA, which was used as gold standard;

**Exclusion criteria**

4. Contraindications to MRI or DSA;
5. Incomplete imaging data or motion artifacts on TOF MRA or TOF-CE MRA.

### Treatment and follow-up DSA

A baseline digital subtraction angiography (DSA) and 3D rotational angiography of the cerebral vasculature were performed in each case prior to treatment. Endovascular treatment and DSA imaging were performed on a Siemens Artis Q or a Philips Azurion biplanar system.

In this study, only chromium-cobalt (Cr-Co) structure FDs (Pipeline Flex with Shield technology, Medtronic, Irvine, CA, USA, or Surpass Evolve, Stryker Neurovascular, Fremont, CA, USA) were used. The flow diverter device was then positioned to cover the entire length of the aneurysm, under fluoroscopic guidance, ensuring alignment with the natural curvature of the parent vessel. Following deployment, a control cone-beam CT (CB-CT) intra-arterial angiogram was performed in all cases to assess the position of the device, the wall apposition, and confirm the adequacy of aneurysm coverage, with a diluted contrast medium (30% contrast agent, 70% normal saline), administered at a flow rate of 1.5 mL/s over a duration of 20 s.

Follow-up DSAs with CBCT were performed to assess residual or recurrent aneurysm and potential braid changes. The follow-up DSA performed at 12 months consisted of subtracted and unsubtracted series with manual contrast injection intra-arterially, as well as an intra-arterial flat-panel detector-CT scan. The injection technique during follow-up was identical to that used during the treatment phase.

### Imaging

MRI was performed on a 3 Tesla (Skyra; Siemens, Erlangen, Germany) scanner using a 64-channel head coil (Siemens, Erlangen, Germany). Participants were scanned in supine position (head first) with a standard clinical protocol for aneurysma investigation. The clinical protocol consisted of pre-contrast native 3D T1, 2D transversal DWI and T2, 3D TOF MRA and TOF-CE MRA.

Native T1-weighted imaging was used for the visual assessment of the aneurysm, particularly to evaluate the presence and extent of intra-aneurysmal thrombosis, due to the signal alterations caused by methemoglobin on native T1-weighted imaging.

Native T1 was measured with the magnetization-prepared rapid gradient echo (MPRAGE, Siemens) using the following imaging settings: transversal field of view (FOV) of 220 × 200 mm measured with a 3D acquisition block containing 40 slices and 6 slabs, image size of 384 × 384 voxels, voxel size of 0.9 × 0.9 × 0.5 mm, slice gap of 0.1 mm, 3.69 ms echo time (TE), 21 ms repetition time (TR), and compressed sensing acceleration factor of 7. Total measurement time was 5:21 min.

TOF MRA and TOF-CE MRA were measured with the 3D gradient echo Time-of-Flight (TOF, Siemens) sequence: transversal FOV of 220 × 220 mm measured with a 6 slabs of 3D acquisition blocks with 40, image size of 384 × 384 voxels, voxel size of 1.0 × 1.0 × 1.0 mm, TE = 3.69 ms, TR = 21 ms and compressed sensing acceleration factor of 7. Total measurement time was 4:18 min.

### Follow-up DSA imaging

The follow-up DSA performed at 12 months consisted of subtracted and unsubtracted series with manual contrast injection selectively in the treated artery, as well as of an intra-arterial flat-panel detector-CT scan using an automated injector with a diluted contrast medium (30% contrast agent, 70% normal saline), administered at a flow rate of 1.5 mL/s over a duration of 20 s.

### Image analysis

Image analysis was performed using DeepUnity Software, a PACS viewer used for post-processing and detailed evaluation of medical images. Two independent neuroradiologists, blinded to clinical outcomes, patient identity, prior imaging, and other modalities, evaluated the images for residual aneurysms and in-stent stenosis. The datasets were assessed over a period of one week, and discrepancies were resolved by consensus. For quantitative assessment, the largest cross-sectional area of each aneurysm sac was measured either on axial T1 or T2 images.

The presence or absence of residual aneurysm filling was assessed in TOF images. In-stent stenosis was graded using the following categories: “yes,” “no,” “possible,” and “unable to assess.” These assessments were crucial for understanding the impact of the Flow-Diverter on both the aneurysm and surrounding arterial flow. Additionally, our results were compared with DSA findings, which served as the gold standard for evaluating residual aneurysm filling and in-stent stenosis.

**Fig. 1:**
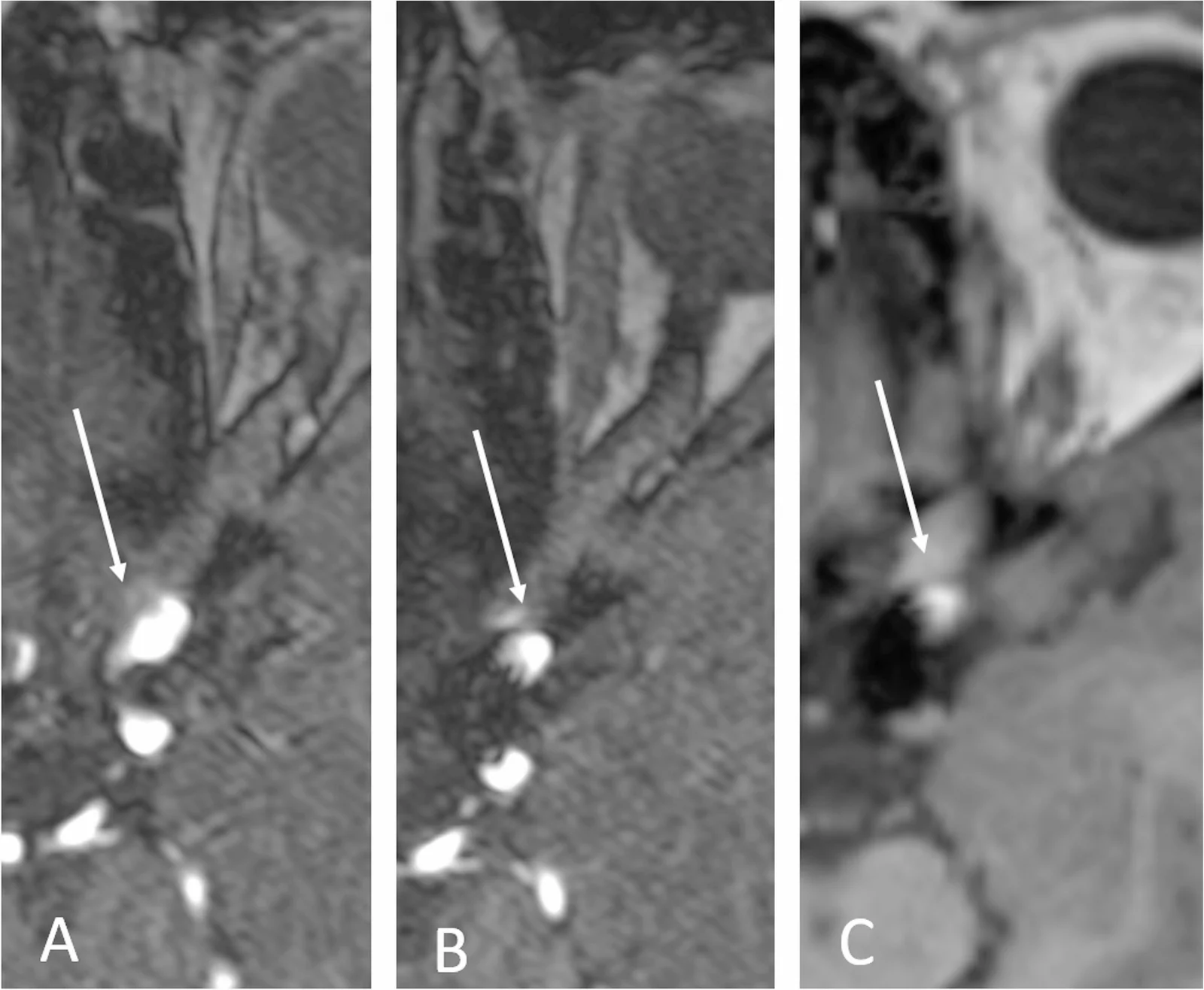
Imaging of a left supraclinoid aneurysm pre- and post-flow diverter treatment. **A** Pre-interventional TOF-MRA showing a left supraclinoid aneurysm with the aneurysm sac highlighted by an arrow. **B** Post-interventional TOF-MRA after Flow-Diverter treatment, demonstrating residual signal within the aneurysm sac. **C** T1-weighted native image, revealing a hyperintense signal at the same location, indicating thrombus formation. The differentiation between residual aneurysm filling (as seen in TOF-MRA) and methemoglobin (suggested by the hyperintense signal on T1) was made based on the T1 and TOF imaging comparison

The differentiation between TOF MRA hyperintensity and T1 methemoglobin hyperintensity was primarily based on T1-weighted imaging. Methemoglobin, indicative of thrombus formation, presents as a hyperintense signal on T1 images due to its paramagnetic properties. The contrast between T1 and TOF images allowed for clear distinction: if the hyperintense signal was present on T1-weighted imaging and absent or weaker on TOF MRA, it was considered to be due to methemoglobin. If the signal appeared in the TOF MRA but was not associated with a corresponding T1 hypersignal, it was considered as residual aneurysm filling (Fig. 1).

Diagnostic accuracy, sensitivity, and specificity were calculated for TOF MRA and TOF-CE MRA relative to DSA.

### Ethical Considerations

The study protocol was approved by the Regional Ethics Coimmitte (KEK), with ethical application number 2022 − 00422. The study conforms to the ethical guidelines of the Declaration of Helsinki.

Informed consent was obtained from all participants or their legal guardians.

## Results

**Fig. 2:**
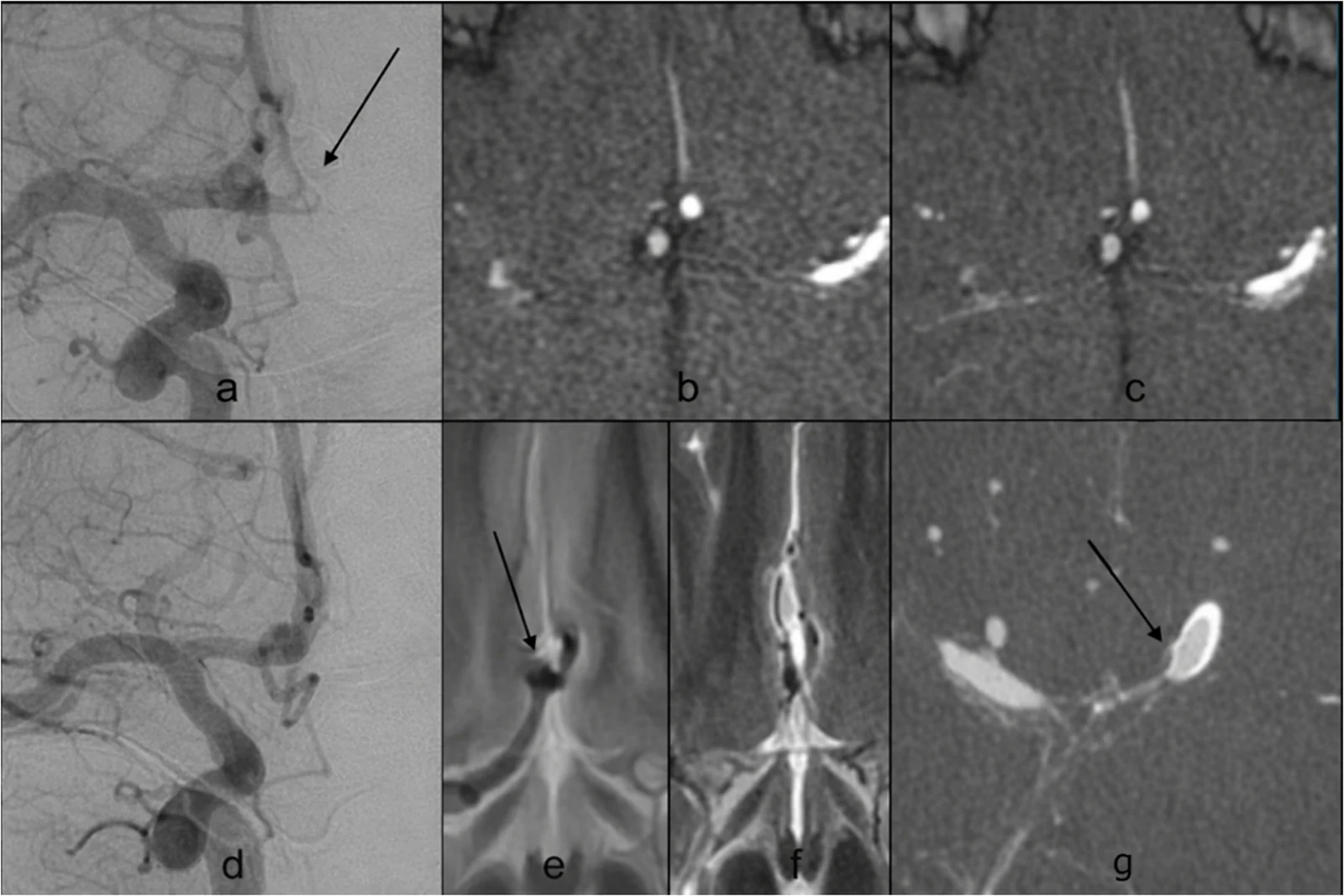
Complete occlusion of an Acom aneurysm of the left internal carotid artery (ICA) 12 months after endovascular treatment using a flow diverter and coils, as seen in follow-up control. **a** DSA before treatment – Pre-treatment DSA showing the Acom aneurysm. **b** TOF-MRA 12 months after treatment – TOF-MRA showing complete occlusion of the Acom aneurysm. **c** TOF-CE 12 months after treatment – TOF-CE showing the occluded Acom aneurysm with no residual filling. **d** DSA 12 months after treatment – DSA after 12 months, confirming complete occlusion of the Acom aneurysm with no residual flow. **e** T2 before treatment – T2-weighted MRI before treatment, showing a flow-void in the Acom aneurysm **f** T2 12 months after treatment – T2-weighted MRI 12 months after treatment, showing a flow-void in the shrunk aneurysm. **g** Vaso-CT 12 months after treatment – Vaso-CT showing the occluded Acom aneurysm and confirming complete closure after 12 months

**Fig. 3:**
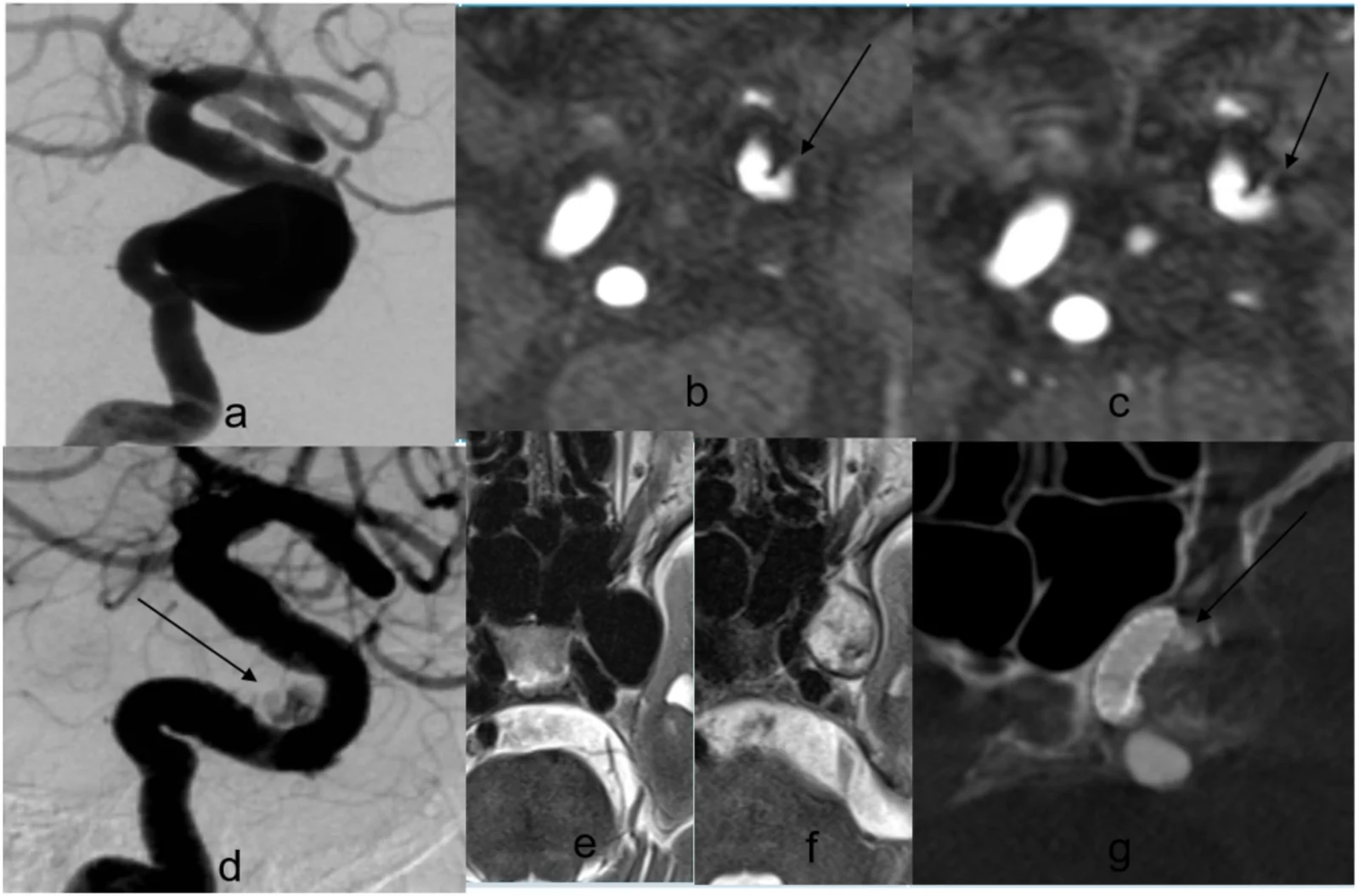
Incomplete occlusion of a cavernous segment aneurysm of the left ICA at 12-month follow-up after endovascular treatment with a flow diverter. **a** DSA before treatment shows a large aneurysm in the cavernous segment of the left ICA. **b** TOF-MRA 12 months after treatment demonstrates minimal residual perfusion at the base of the treated aneurysm. **c** TOF-CE-MRA 12 months after treatment also reveals a small residual perfusion at the aneurysm base. **d** DSA at 12-month follow-up confirms the persistent residual perfusion. **e** T2-weighted MRI before treatment shows a flow void corresponding to the aneurysm. **f** T2-weighted MRI 12 months after treatment shows a shrunken aneurysm. **g** Vaso-CT 12 months after treatment again demonstrates minimal residual perfusion at the aneurysm base

Of the initial 143 patients, 70 were excluded from the analysis. The most common reasons were patients not consenting to the study, incomplete or missing imaging datasets, non-diagnostic image quality due to motion or other artifacts, and follow-up examinations performed at outside institutions at the request of the patients. Consequently, a total of 73 patients fulfilled all inclusion criteria and were available for statistical analysis (Figs. 2 and 3).

The demographics of the cohort included a mean age of 55 years (± 34–78 years). Forty-five patients (62%) were female, and twenty-eight (38%) were male. Most aneurysms were located in the anterior circulation (60%), including the internal carotid artery (28%) and anterior communicating artery (AcomA, 32%). The remaining 40% were found in the posterior circulation, such as the basilar artery (BA) and posterior communicating artery (PcomA).

At 12 months residual aneurysm filling was identified in 15 out of 73 cases (20%) using DSA. 14 cases were detected on TOF-MRA, missing 1 case (false negative), while 13 cases were detected on TOF-CE MRA, missing 2 cases. The minimal aneurysm filling adjacent to the stent was visible in DSA in all cases; the TOF-MRA slightly overestimated residual filling in 3 cases and TOF-CE showing overestimation in 2 cases. Regarding in-stent stenosis, DSA identified 10 cases, whereas TOF-MRA detected 8 cases, missing 2 cases of mild stenosis, and TOF-CE detected 9 cases, missing 1 case. Both modalities faced limitations in clearly defining the severity of stenosis, particularly for smaller parent vessels.

Aneurysm occlusion grades were evaluated using DSA as the reference standard. Among the incompletely occluded aneurysms, TOF-MRA correctly classified 14 cases but overestimated occlusion in 1, whereas TOF-CE-MRA correctly classified 13 and overestimated in 2.

Overall, TOF-MRA demonstrated high sensitivity (96%) but moderate specificity (66%), while TOF-CE exhibited slightly lower sensitivity (91%) but somewhat specificity (73%). However, these differences were not statistically significant.

Mann-Whitney U-test confirms a highly significant reduction of the aneurysm size (*p* = 0.0001), supporting the effectiveness of the treatment in reducing aneurysm size at 1 year.

Statistical analysis showed substantial inter-reader agreement for both imaging modalities (kappa = 0.68) in evaluating residual aneurysm filling and in-stent stenosis.

## Discussion

In this study, we compared the diagnostic performance of TOF-MRA and TOF-CE MRA in the follow-up of aneurysms treated with flow diverters. Based on the hypothesis that contrast-enhanced (CE) imaging may improve the diagnostic value of TOF-MRA, we investigated the diagnostic performance of TOF-MRA with and without CE, compared to Digital Subtraction Angiography (DSA). The findings reveal that while both MRI-based modalities are valuable as non-invasive tools, TOF-CE MRA did not significantly improve diagnostic performance over standard TOF-MRA, particularly in evaluating aneurysm occlusion and detecting in-stent stenosis in complex or large aneurysms. This indicates that the addition of CE to TOF-MRA does not provide substantial improvements in diagnostic accuracy for these cases.

TOF-MRA demonstrated high sensitivity (96%) in detecting residual aneurysms, while TOF-CE MRA offered slightly improved specificity (73% vs. 66%). However, both sequences were prone to overestimating aneurysm remnants, particularly in larger aneurysms (> 10 mm). This overestimation may be attributed to the challenging anatomy of large aneurysms and the complex flow dynamics, which can result in artificial signal enhancement, particularly in regions with turbulent flow.

Additionally, susceptibility artifacts can affect the accuracy of the measurements, especially in areas with metal implants or areas close to the bone, leading to a distorted signal. This trend is consistent with previous work on MRI–DSA agreement and measurement performance in treated aneurysms [18]. TOF-CE did not improve the diagnostic performance with regard to in-stent stenosis. Although DSA identified 10 cases, TOF-MRA detected 8 and TOF-CE MRA detected 9 cases, with a tendency to miss mild stenoses, especially in small parent vessels. Published recommendations and reference standards reinforce the limitations of non-invasive imaging for subtle stenoses in the presence of metallic implants [19]. Artifact-induced pseudo-stenosis was observed in 36% of TOF MRA and 31% of TOF-CE MRA images in our study—changes not present on DSA.

We observed that diagnostic accuracy varied according to the aneurysm size. Larger aneurysms showed increased misclassification rates, which aligns with reports addressing volumetric and hemodynamic behavior after flow diversion [20]. In our cohort, aneurysms > 10 mm exhibited higher rates of overestimation of aneurysm occlusion on MRI compared to DSA, whereas aneurysms < 3 mm were evaluated more accurately.

Regarding treatment outcomes, our study found a 13% average reduction in aneurysm size one year post-treatment (*p* = 0.0001). While this result supports the efficacy of flow diverters in promoting progressive aneurysm thrombosis and shrinkage, it was not a primary study aim and is presented as a secondary finding. This aligns with quantitative MRI volume analyses and longer-term clinical follow-up, which show relief of mass effects [21].

The observed differences in sensitivity and specificity between TOF-MRA and TOF-CE MRA can be explained by their distinct imaging principles. Non-contrast TOF relies on flow-related enhancement, which can be prone to signal loss in areas of slow or turbulent flow, particularly near the stent struts or regions with complex flow dynamics. This can lead to overestimation of small residual fillings, resulting in higher sensitivity but also increasing the likelihood of false positives, thus lowering specificity. In contrast, TOF-CE provides a more homogeneous intravascular signal that is less dependent on flow velocity, reducing false positives and improving specificity. However, the increased background signal after contrast injection can obscure subtle residual filling, leading to reduced sensitivity. Additionally, susceptibility artifacts caused by metal stents or proximity to bone may distort the signal, contributing to inaccuracies in the measurement of aneurysm remnants. These complementary strengths and weaknesses account for the discrepancies observed between TOF and TOF-CE in our cohort.

Importantly, DSA remains the gold standard for detecting aneurysm filling, stent patency, and device-related complications. However, it has inherent limitations—it cannot assess ischemic or hemorrhagic complications, tissue remodeling, or brain parenchymal changes. MRI complements DSA by enabling detection of silent infarctions, evaluating hemorrhagic transformation, and measuring changes in aneurysm morphology. It provides a broader perspective on treatment response and post-procedural safety.

Moreover, in rare instances, aneurysm growth has been observed after flow-diverter implantation. Such enlargement despite complete device deployment has been described, potentially due to ongoing thrombus formation or inflammatory remodeling [22, 23]. These cases underscore the value of MRI as a surveillance tool for atypical outcomes—especially for partially thrombosed or giant aneurysms, where DSA may not detect dynamic morphological changes.

Fiehler et al. (2024) suggest standardizing the imaging evaluation for the follow-up of flow diverter treatments, particularly in relation to the O’Kelly-Marotta Classification (OKM) for aneurysm occlusion. They highlight that while DSA remains the gold standard for evaluating aneurysm occlusion and in-stent stenosis, non-invasive methods like TOF-MRA can complement DSA, especially for assessing aneurysms without contrast agents or radiation exposure [24]. Similarly, we find that a combination of DSA and MRA is an effective approach for follow-up after flow diverter treatment.

The type of flow diverter used can influence the appearance of artifacts on magnetic resonance angiography (MRA). Studies have shown that different flow diverter materials, such as chromium-cobalt (Cr-Co) versus nitinol (NiTi), can produce varying artifact sizes and intensities, potentially affecting the diagnostic accuracy of imaging techniques like TOF-MRA and TOF-CE MRA. Specifically, Cr-Co devices have been associated with larger artifacts and lower specificity compared to nitinol devices, which may impact the ability to accurately assess residual aneurysm filling or in-stent stenosis [25]. However, in our study, we used only Cr-Co alloy devices, and the results may not apply one-to-one to NiTi structures.

However, while TOF-CE MRA showed slightly improved specificity, our results demonstrated that it did not provide substantial additional information compared to non-contrast TOF-MRA. Given the added time, cost, and contrast use, routine use of TOF-CE MRA appears unnecessary. TOF-MRA alone is sufficient for evaluating most post-treatment aneurysms, making it a more practical and cost-effective choice for standard follow-up protocols.

This study has several limitations. First, it is retrospective in nature and was conducted at a single academic center, which may limit the generalizability of the findings. Second, the relatively small sample size (73 patients) limits statistical power, especially for subgroup analyses of large versus small aneurysms. Additionally, a considerable proportion of the initial cohort (70 out of 143 patients) was excluded, which could lead to selection bias. Many of the excluded patients had follow-up assessments performed at other centers or abroad, limiting the availability of complete imaging data. Third, although imaging was performed using a standardized protocol, technical variations such as patient motion, stent orientation, or susceptibility artifacts may have influenced image quality and diagnostic accuracy. Fourth, the evaluation of in-stent stenosis and residual aneurysm filling was based on consensus readings of two neuroradiologists, which introduces some degree of subjectivity despite substantial inter-reader agreement (kappa = 0.68), reflecting variability in the assessment due to factors such as image artifacts and the complexity of the lesions.

## Conclusion

While TOF-CE MRA showed slightly improved specificity, it did not provide significant additional value compared to TOF-MRA for the follow-up imaging assessment of aneurysms treated with flow diverter stents. However, subtle differences cannot be entirely excluded given the sample size. When it comes to the assessment of in-stent stenosis and unclear residual aneurysm filing, DSA with CB-CTA still cannot be replaced by MRI. As such, the combination of DSA and MRA remains the most effective strategy for post-treatment follow-up of intracranial aneurysms treated with flow diverters, the first assessing residual filling, stent structure and in stent stenosis, while the second is best to assess aneurysm size changes.

## Data Availability

No datasets were generated or analysed during the current study.
